# The Roles of Histone Post-Translational Modifications in the Formation and Function of a Mitotic Chromosome

**DOI:** 10.3390/ijms23158704

**Published:** 2022-08-05

**Authors:** Marco A. Andonegui-Elguera, Rodrigo E. Cáceres-Gutiérrez, Alejandro López-Saavedra, Fernanda Cisneros-Soberanis, Montserrat Justo-Garrido, José Díaz-Chávez, Luis A. Herrera

**Affiliations:** 1 Unidad de Investigación en Cáncer, Instituto de Investigaciones Biomédicas-Universidad Nacional Autónoma de México, Instituto Nacional de Cancerologia, San Fernando 22, Sección XVI, Tlalpan, CDMX, Mexico City 14080, Mexico; 2 Instituto Nacional de Medicina Genómica, Periferico Sur 4809, Arenal Tepepan, Tlalpan, Mexico City 14610, Mexico

**Keywords:** histones, mitosis, centromere, kinetochore, chromosome condensation, nickel, arsenic

## Abstract

During mitosis, many cellular structures are organized to segregate the replicated genome to the daughter cells. Chromatin is condensed to shape a mitotic chromosome. A multiprotein complex known as kinetochore is organized on a specific region of each chromosome, the centromere, which is defined by the presence of a histone H3 variant called CENP-A. The cytoskeleton is re-arranged to give rise to the mitotic spindle that binds to kinetochores and leads to the movement of chromosomes. How chromatin regulates different activities during mitosis is not well known. The role of histone post-translational modifications (HPTMs) in mitosis has been recently revealed. Specific HPTMs participate in local compaction during chromosome condensation. On the other hand, HPTMs are involved in CENP-A incorporation in the centromere region, an essential activity to maintain centromere identity. HPTMs also participate in the formation of regulatory protein complexes, such as the chromosomal passenger complex (CPC) and the spindle assembly checkpoint (SAC). Finally, we discuss how HPTMs can be modified by environmental factors and the possible consequences on chromosome segregation and genome stability.

## 1. Introduction

In eukaryotic cells, the DNA is bound to histone and non-histone proteins in a complex known as chromatin. In total, 147 base pairs of DNA are wrapped on a histone octamer formed by two copies of each of the core histones (H2A, H2B, H3, and H4). This unit is known as a nucleosome. Between nucleosomes, there are stretches of DNA bound to the linker histone H1 [1]. Core histones are constituted by a largely alpha-helical C-terminal domain, which is involved in the formation of the octamer and the DNA binding. The N-terminal region of core histones is organized in an unstructured tail. These tails and the C-terminal of H2A are the sites where most post-translational modifications are found [1,2]. Histones, like other proteins, can be modified in specific residues with the addition of different molecules; they can be acetylated, methylated, phosphorylated, and ubiquitinated, among other modifications. These modifications can change their affinity for DNA or can be “read” by other proteins and recruit specific factors to the nucleosome. Lysine acetylation was the first HPTM described. It opens condensed chromatin by promoting a charge neutralization in histones. Acetylated histones form a more relaxed chromatin which facilitates the recruitment of transcription machinery. Moreover, lysine acetylation is recognized by the bromodomain of several proteins that regulate gene expression. Lysine acetylation is also involved in DNA repair. This mark is deposited by the histone acetyltransferases (HATs) and removed by the histone deacetylases (HDACs) [3,4].

Histone lysines and arginines can be methylated by enzymes that use S-adenosyl-L-methionine as a methyl donor. Lysines can be mono-, di-, or trimethylated, while arginines can be mono- or dimethylated. The role of lysine methylation in transcriptional regulation is well known. Lysine methylation is “read” by chromodomain-containing proteins, which can promote or repress transcription [5]. Lysine methylation is removed by lysine-specific demethylases (LSDs) or members of the Jumonji family of demethylases. On the other hand, the arginine methylation of histones is also associated with the regulation of gene expression [2]. The effect of histone methylation can differ depending on the position of the methylated residue and its level (mono-, di-, or trimethylated) [6,7]. Histone phosphorylation is an important mark for the DNA damage response (DDR). Histone phosphorylation is also associated with transcription regulation [2,8]. Finally, histone ubiquitination is another mark that has been associated with transcription regulation and DDR [9]. It has been proposed that histone modifications in the same histone tail or nearby tails from different histones can constitute a “code” that is interpreted to perform different functions in chromatin. Although the regulation of gene expression and DDR using histone HPTMs has been extensively studied, the role of histone PTMs in chromosome segregation has recently been revealed.

In addition to transcriptional regulation and DNA repair, HPTMs can play other structural and functional roles along the cell cycle. Particularly in mitosis, HPTMs are involved into the chromatin condensation, forming visible chromosomes under the microscope. Chromosome condensation is necessary to facilitate the transport and separation of genetic material to daughter cells. In this process, chromatin reduces its length by up to four orders of magnitude, significantly modifying its structure and physical properties [10]. Each chromosome harbors a region known as centromere, whose identity is defined by the presence of the distinctive H3 histone variant CENP-A, as well as several accompanying HPTMs [11]. The centromere is important because a multiprotein complex known as kinetochore is assembled onto that region, and it binds microtubules to align the chromosomes [12]. In addition, specific HPTMs also regulate the spindle assembly checkpoint (SAC) and the activity of the chromosomal passenger complex (CPC). The SAC checks the union between microtubules and kinetochores and stops mitosis until all chromosomes are attached to microtubules from the mitotic spindle. On the other hand, the CPC corrects kinetochore–microtubule attachment errors and participates in the contractile ring formation during cytokinesis [13,14]. Chromatin and specific HPTMs are involved in several of these processes during mitosis.

Remarkably, HPTMs are not only modified by cellular intrinsic factors but also by extrinsic environmental factors. For example, environmental pollutants can also modify HPTMs and disturb some of the mechanisms they are involved in [15]. Chromosome segregation is one potential mechanism that can be disturbed by environmental pollutants since histone PTMs are involved in its regulation.

In this review, we focus on the role of histone PTMs in mitosis, from chromosome condensation to the regulation and activity of the centromere and kinetochore. We also discuss how environmental factors could modify histone PTMs and disturb chromosome segregation.

## 2. Chromosome Condensation

During mitosis, chromatin is condensed to form discrete units known as mitotic chromosomes. Condensation facilitates the segregation of the exceptionally long DNA molecules, which form chromosomes. Many factors have been associated with chromosome condensation, but the specific mechanism whereby chromatin is condensed in a mitotic chromosome is not well understood. In an in vitro model of chromosome condensation, six factors were shown to be essential: core histones, nucleoplasmin, Nap1, FACT, topoisomerase II (topo II), and condensin I [16]. Although these purified factors could form mitotic chromosomes, it is unknown if condensation is achieved in a normal way because compaction was not quantified in this assay.

**Figure 1:**
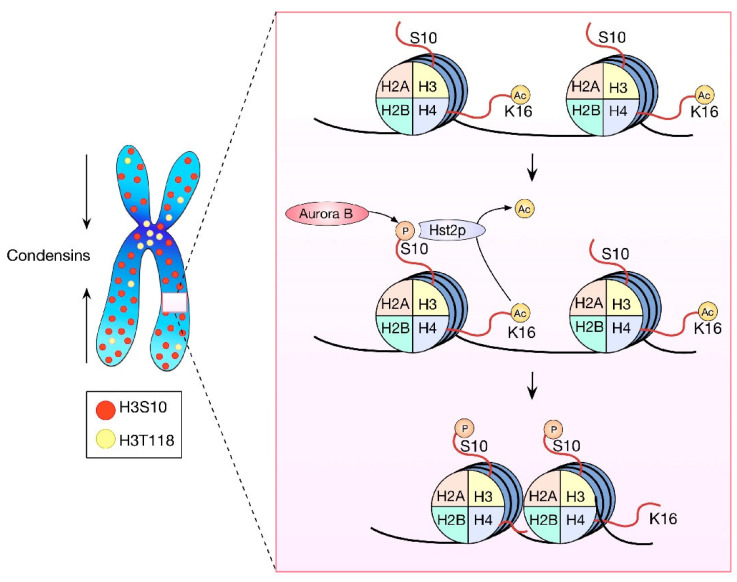
HPTMs in chromosome condensation. Condensins participate in the axial shortening of chromosomes while histone modifications allow the local compaction. H3 is phosphorylated in the serine 10 along all the chromosomes. This modification recruits Hst2p, which deacetylates H4K16 by inducing local nucleosome joining and local chromatin compaction. H3T118 also participates in chromosome condensation, and its distribution is preferentially in the pericentromeric chromatin, but its function is still unknown.

It is known that nucleosomes are essential for chromosome condensation, although the role of histone modifications in this mechanism is in debate. The phosphorylation of histone H3 at serine 10 (H3S10ph) using the Aurora B kinase is noticeable since prophase, and it has been associated with chromosome condensation [17,18]. However, several reports have challenged the role of H3S10ph in chromosome condensation. The mutation of serines 10 and 28 of H3 does not alter chromosome condensation in yeast [19]. Furthermore, chromosome condensation is not significantly modified in Drosophila and human cells when Aurora B is inhibited [20,21]. Finally, incubation with a hypotonic solution can promote H3S10 dephosphorylation in HeLa cells without an observable difference in chromosome structure in comparison with cells that retain the H3S10ph mark [22]. However, some data support the role for H3S10ph in chromosome condensation. There is a cascade of histone modifications during mitosis that promotes chromatin compaction via the interaction between adjacent nucleosomes in yeast. H3S10ph recruits Hst2p (a histone deacetylase), which in turn deacetylases the lysine 16 of H4 (H4K16ac). The deacetylation of H4K16 allows the contact between the N terminus of H4 and the dimer interface of H2A-H2B in the adjacent nucleosome, promoting chromatin compaction (Figure 1) [23]. Moreover, Barral et al. have demonstrated the role of H3S10ph in chromatin compaction during mitosis but not in the axial shortening of chromosomes in yeast [24]. It is important to note that these studies have been performed in yeast with relatively small chromosomes. A study with mammalian chromosomes showed that the highest level of compaction occurred during anaphase and that it depends on axial shortening mediated by Aurora B activity. However, whether such activity depended on H3S10ph or another substrate of Aurora B was not established [25].

Although several reports have raised controversy about the specific role of H3S10ph in chromosome condensation, it is important to note that most of these studies are only qualitative, and chromosome condensation is visually evaluated as the formation of a typical chromosome without quantifying the level of condensation. Recently, fluorescence-based techniques have been used to measure chromosome condensation by quantifying the distance between two fluorescent marks in a chromosome (axial shortening) or the measurement of the fluorescence level of a region where lower fluorescence is associated with a more compacted chromatin due to a quenching effect (chromatin compaction) [24,26]. Using these techniques, it has been demonstrated that chromosomes are compacted by two independent pathways, first by local compaction, which is due to changes in histone modifications, and then by axial shortening, which depends on the action of Condensins.

The function of Aurora B kinase in the formation of mitotic chromosomes has been demonstrated in different studies. In chicken DT40 cells, it was demonstrated that a factor (RCA regulator of chromosome architecture) maintains chromosome structure independently of condensins [27]. This factor is inhibited by Repo-Man-PP1γ, a complex whose phosphatase activity eliminates H3 phosphorylation by Aurora B [28]. There is a negative feedback between Repo-Man-PP1γ, and Aurora B. Repo-Man-PP1γ removes the H3T3ph tag required for CPC recruitment, whereas Aurora B phosphorylates Repo-Man, preventing PP1γ recruitment to the chromosome [28,29]. Although the role of Aurora B activity in chromosome condensation is clear, it has not been shown to depend exclusively on its kinase activity on the H3S10 residue. Repo-Man-PP1γ was shown to participate mainly in the dephosphorylation of H3T3ph and H3T11ph [28].

The aurora A-dependent phosphorylation of threonine 118 on H3 (H3T118ph), which occurs at the histone DNA interface, is present in mitotic chromosomes from prophase until metaphase and is also associated with chromosome condensation in metazoans. The expression of the phosphomimetic mutant H3 T118I or Aurora A overexpression cause the shortening and widening of chromosome arms. Moreover, the mutant H3 T118I diminished the Condensin I association with chromatin through an unknown mechanism (Figure 1) [30]. On the other hand, in an in vitro model, the N terminus of H2B was shown to be essential to achieve chromosome condensation, and the N terminus of H2A and H4 was also important to condensation, but to a lesser extent [31].

Changes in the methylation of the H3K9 residue during mitosis have also been found. In mammalian cells, H3K9 methylation levels decrease during mitosis (mono-, di-, and trimethylation), especially for H3K9Me3 [32,33,34]. However, some methylation levels at H3K9 are necessary for segregation because their inhibition causes defects in chromosome segregation [34]. By using FRET biosensors, it was demonstrated that H3S10 phosphorylation causes H3K9Me3 to decrease. Furthermore, this study showed that the H3K9Me3 decrease is associated with the dissolution of heterochromatin structures, which they propose is necessary for proper global chromosome compaction in mitosis [32]. Moreover, the decrease in H3K9Me3 coincides with an increase in global histone H1 phosphorylation. However, the role of this change in chromosome formation and segregation is unclear [33].

## 3. Histone Modifications, CENP-A Incorporation, and Kinetochore Function

The centromeric chromatin is recognized to contain a histone modification pattern different from those classically related to active or repressive chromatin. Karpen and Sullivan coined the term “centrochromatin” to refer to this specialized type of chromatin [35,36]. However, the real diversity of HPTMs present in this region and their specific roles are only starting to be uncovered. Recent data show that the centromeric chromatin context is a key determinant of proper mitotic progression [37], since it bears HPTMs that promote both the correct function of the inner centromere, through the regulation of the chromosomal passenger complex and the assembly of the kinetochore through the control of CENP-A centromeric incorporation.

### 3.1. HPTMs and the Chromosomal Passenger Complex

The CPC is composed of Survivin, Borealin, INCENP, and the kinase Aurora B, and it is involved in the control of the mitotic chromosome structure, spindle assembly checkpoint regulation, microtubule–kinetochore union, the formation of the mitotic spindle, and cytokinesis [38,39]. The mechanism by which the CPC is located to chromosome arms to induce mitotic chromosome structure has been elusive until recently when the asymmetrical dimethylation of arginine 2 was detected on H3 in human cells [40]. H3R2me2a, brought about by the methyltransferase PRMT6, is abundant from the S phase until the M/G1 transition. This HPTM was shown to participate in the recruitment of the CPC to chromosome arms during the late G2/early prophase. The relation between H3R2me2a, the CPC, H3S10ph, and mitotic chromosome condensation was determined by tomographic microscopy. In the same study, the authors reported that while the cohesion of both mitotic chromosome arms and centromeres rely upon H3R2me2a, the former depends on CPC recruitment and H3S10ph, while the latter is mediated by the centromeric localization of Shugoshins 1 and 2 [40].

**Figure 2:**
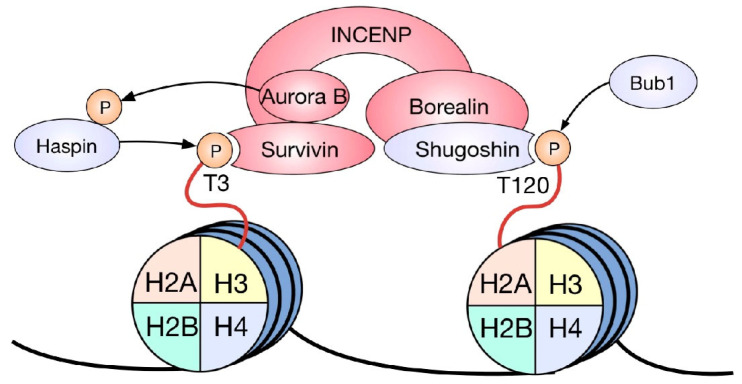
HPTM-dependent CPC recruitment. H3T3 and H2AT120 phosphorylations, by Haspin and BUB1, respectively, are two key HPTMs necessary for the recruitment of the CPC to centromeres. The coincidence of these marks on mitotic chromosomes specifies the site of CPC localization. Survivin binds to H3T3ph, conveying the other components of the complex, while Shugoshin binds to H2AT120, acting as an adaptor between this mark and Borealin. A phosphorylation-dependent positive feedback loop occurs between Aurora B and Haspin kinases, reinforcing the CPC recruitment pathway.

In terms of SAC regulation, the CPC is responsible for the destabilization of incorrect kinetochore–microtubule attachments [41]. Unattached kinetochores result in the activation of the SAC, with a consequential mitotic arrest. CPC function depends on its correct localization in metaphase, which in turn relies upon two mitosis-specific histone marks. During metaphase, this complex is recruited to the inner centromere by the phosphorylation of histone H3 at threonine 3 and the phosphorylation of histone H2A at threonine 120 [42]. These marks have a different distribution throughout mitotic chromosomes: H3T3ph is observed along the entire arms of the chromosome, proximal to the site where both sister chromatids contact each other, while H2AT120ph is found along the axis that connects the kinetochores of two sister chromatids [42]. Interestingly, it has been shown that the CPC is recruited to the intersection of both histone phosphorylations, i.e., the inner centromere (Figure 2). The phosphorylation of H3T3 by the kinase Haspin [43] is recognized and bound by a pocket in the BIR domain of Survivin [44], while H2AT120ph recruits the CPC through Shugoshin1. This protein directly binds to the phosphorylated residue and functions as an adaptor by contacting Borealin, thus tethering the CPC to the site of H2AT120ph in an indirect manner [45]. Furthermore, inhibiting the histone phosphorylations that recruit the CPC to the inner centromere during metaphase by depleting the kinases responsible for them or preventing their recognition with the use of specific antibody microinjection causes the delocalization of the CPC and consequently has detrimental effects on mitotic progression [42,43].

### 3.2. HPTMs and CENP-A Centromeric Incorporation

On the other hand, CENP-A, whose recruitment is also highly dependent on HPTMs, is the most critical protein of kinetochore assembly. It is a histone H3 variant and the distinctive epigenetic element of active centromeres [11]. CENP-A is the interface between the centromere and the constitutive centromere-associated network (CCAN), a large protein complex that directly tethers to microtubules during chromosome segregation [46]. Accordingly, CENP-A recruitment is tightly regulated, and the same amount is incorporated into the centromere in every cell cycle in normal conditions [11].

CENP-A cellular production occurs during the late synthesis/G2 phase of the cell cycle. However, its centromeric incorporation is prevented until the next G1 by inhibitory phosphorylations on HJURP, Mis18BP1, and CENP-A itself (see below), mediated by the CDK1 and CDK2 kinases. Instead, the nucleosome gaps in the centromeric chromatin resulting from the distribution of CENP-A among the old and newly synthesized DNA during the S phase are filled with histone H3.1- and histone H3.3-containing nucleosomes. In the next G1 phase, histone H3.3-containing nucleosomes are exchanged for nucleosomes containing newly synthesized CENP-A [47,48,49]. Notably, the machinery responsible for CENP-A incorporation ultimately relies on the coordination of several HPTMs on different histones within specific time frames. Such modifications have been shown to occur on histones H2B, H3, and H4, as well as CENP-A itself.

Histone H3 from nucleosomes adjacent to CENP-A nucleosomes was recently purified and analyzed by mass spectrometry. This allowed the identification of a complex mixture of modifications, including the acetylation of residues K14, K18, and K23, and the mono-, di-, and trimethylation of residues K4, K9, K27, and K36 [35]. Of note, the presence of CENP-A in centromeric nucleosomes is mutually exclusive with that of canonical H3 [50,51,52,53]. However, CENP-A-containing nucleosomes are interspersed with those containing H3 [36]. Therefore, CENP-A-associated chromatin contains histone H3 from nucleosomes neighboring those that do contain CENP-A. While centromeric H3 is enriched in methylation, its acetylation was found to be rather scarce [35]. In agreement with this, H3K9 trimethylation impedes centromeric nucleosome turnover while H3K9 acetylation permits it, and this process occurs exclusively during late mitosis/early G1 [54]. Therefore, the observation that the centromere bears low acetylation levels seems to be a result of the temporal regulation to which this HPTM is subjected. Accordingly, centromere acetylation takes place only during a short time frame at such a point of the cell cycle, and this results in low levels of centromere acetylation detection in asynchronous cells [54].

Further support for the importance of centromeric chromatin HPTMs comes from work in the Larionov, Earnshaw, and Masumoto laboratories. Using human artificial chromosomes (HACs), they developed a strategy to evaluate the function of chromatin modifiers localized directly on the centromere [55]. Their studies revealed that the excess of transcriptional activity formed by transcriptional activators and the excess of heterochromatin formed by transcriptional silencers could affect kinetochore assembly by the improper incorporation of H3K4me2 (associated with active transcription) and H3K9me3 (associated with transcriptional repression), respectively [55]. The overexpression of LSD1, an H3K4 histone demethylase localized directly to the centromere, reduces H3K4me2 presence in this region. Furthermore, the modification of H3K4me2 levels in the centromeric regions impedes the incorporation of CENP-H and CENP-C, HJURP, and CENP-A, therefore hindering correct kinetochore function [56,57].

On the other hand, it has been shown that CENP-A assembly can be achieved at ectopic alphoid DNA regions in several human cell lines. However, these CENP-A arrays are commonly lost, and their loss is accompanied by an increase in the trimethylation of histone H3 at lysine 9 [54]. Interestingly, the abundance of both H3K9me3 and H3K9ac can vary in different cell lines, and the balance between these two marks is associated with the propensity to retain ectopic CENP-A arrays. For instance, the cell line HT1080 has a higher tendency to retain such arrays than HeLa, TIG7, hTERT-BJ1, and U2OS. This is associated with the H3K9me3/H3K9ac ratio, which is lower in HT1080 cells [54]. Furthermore, the depletion of Suv39h1, an H3K9 methyltransferase, increases CENP-A incorporation to the ectopic array, while ectopically targeting acetyltransferases to an alphoid DNA array promotes de novo kinetochore formation in HeLa cells. Thus, the presence of either the methylation or acetylation of H3 at the centromere is determinant in the maintenance of a functional centromere [54].

**Figure 3:**
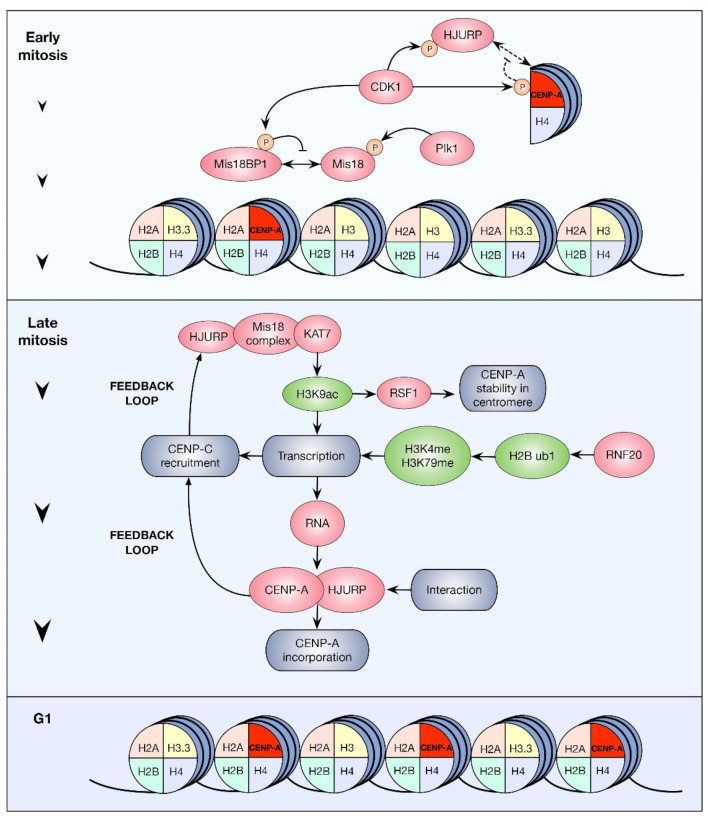
Roles of HPTMs in CENP-A incorporation. In every cell cycle, half of the CENP-A nucleosomes from each centromere are loaded onto the nascent DNA strand during DNA replication. As a result, G2 centromeres show half of the CENP-A nucleosomes that G1 centromeres have. The incorporation of new CENP-A nucleosomes remains inhibited during G2 and early mitosis by specific phosphorylations on histone and non-histone proteins (upper panel). These phosphorylations prevent the formation of key complexes for CENP-A incorporation, such as the Mis18BP1 complex. However, CENP-A nucleosome levels are replenished during late mitosis/early G1. The methylation, acetylation, and ubiquitination of different histones are required during the process (middle and lower panel). This cascade of events involves at least two feedback loops, ultimately leading to increased CENP-A incorporation. HPTMs are depicted in green, proteins and RNA in pink, and processes in grey.

Moreover, centromere acetylation is the result of an interesting cascade of events involving the centromeric localization of the Mis18 complex (Figure 3). Some lines of evidence strongly suggest that the central role of this complex in CENP-A incorporation is the priming of centrochromatin [11]. In human cells, the Mis18 complex, which is composed of hMis18α, hMis18β, and hMis18BP1, is specifically accumulated at the telophase-G1 centromere [58] and promotes the recruitment of the KAT7 histone acetyltransferase, which, in turn, acetylates histone H3 on lysine 14 [59]. The depletion of Mis18 complex subunits has been rescued by the treatment of cells with the histone deacetylase inhibitor trichostatin A [60] and by the specific targeting of chimeric histone acetyltransferases to an exogenous centromere [54]. The acetylation of centrochromatin can elicit changes in the chromatin state in at least three different aspects: it promotes chromatin relaxation, which facilitates the eviction of histone H3-containing nucleosomes and the incorporation of CENP-A-containing nucleosomes; it recruits the remodeling and spacing factor 1 (RSF1), a protein required for the stabilization of CENP-A at the centromeric array [59]; and it also permits centromeric transcription which gives rise to non-coding RNAs derived from alpha satellite, which have been shown to participate in HJURP-mediated CENP-A incorporation to the centromere (Figure 3) [61]. Centromere H3 acetylation is not the only HPTM associated with centromeric transcription around the time of CENP-A assembly. A transient pulse of centromeric H2B monoubiquitination is brought about by the ubiquitin ligase RNF20 in mitosis [62]. This histone modification stimulates the RNA pol-II-mediated transcription of the centromere. Previous work has shown that H2BK120ub1 promotes H3K4 di- and trimethylation through the hSET1 complex [63], and H3K79 methylation mediated by hDOT1L [64]. H3K4me2/3 is related to the transcription start site of active genes, while H3K79me has been found in the body of active genes [65], providing a mechanistic explanation for the observed increase in centromeric transcription. These changes, elicited by H2BK120ub1, decrease histone H3 stability at the centromere, facilitating CENP-A incorporation. Furthermore, inhibiting H2BK120ub1 by the depletion of RNF20 with the use of RNAi is associated with an increase in silencing histone modifications and a decrease in centromeric transcription and histone turnover, thus affecting CENP-A assembly. Naturally, this phenotype has detrimental consequences on chromosome segregation [62].

Another HPTM important for the centromere structure and kinetochore assembly is H4K20me1 [66]. PR-Set7 is the enzyme responsible for H4K20 methylation, and its regulation is cell-cycle-dependent [67]. During the interphase, H4K20me1 is preferentially associated with the 5′ end of actively transcribed genes [68]. However, during late mitosis, H4K20me1 is located on the CENP-A nucleosomes, and this modification occurs after CENP-A incorporation. Recent studies have demonstrated that an increase in PHF8 (an H4K20me1 histone demethylase) inhibits CENP-A-H4K20me1 mature nucleosome formation, reducing CENP-H and CENP-T localization to centromeres and promoting kinetochore assembly defects [66].

On the other hand, the RbAp46/RbAp48 complex recognizes the CENP-A/H4 prenucleosomal dimer and recruits the histone acetyl transferase Hat1, which catalyzes H4K5 and H4K12 acetylation [69,70]. Using RbAp48 depletion, this process was shown to be required for CENP-A deposition. Based on a mutational analysis, the authors of this study also proposed that the failure to neutralize the H4K5- and H4K12-positive charges explains the CENP-A incorporation errors observed in RbAp48-depleted cells. Specifically, they suggested that the acetylation-free H4 tail could interfere with the Mis18 complex–HJURP interaction, therefore hindering proper CENP-A nucleosome centromeric deposition [70].

Post-translational changes on CENP-A itself are also required for its correct incorporation to the centromere. However, a comprehensive model of the roles played by each of the PTMs discovered on this histone variant is difficult to construct due to conflicting experimental evidence.

Although the sequence of the CENP-A N terminus shows poor conservation, the presence of potentially phosphorylatable serines is a conserved feature of the CENP-A N terminus from this domain among diverse eukaryotic species [71]. For example, CENP-A is phosphorylated on serine 7 by Aurora kinases during mitosis. This modified CENP-A has been shown to localize to the contractile ring and participate in the completion of cytokinesis [72]. Interestingly, CENP-A depletion can be rescued by the expression of a mutant CENP-A in which the amino tail was replaced by the canonical H3 amino tail, which also contains two serines, in positions 10 and 28. These residues are known to be phosphorylated during mitosis [73]. CENP-A amino tail phosphorylation was proposed to be necessary for kinetochore function by recruiting 14-3-3 proteins, which would, in turn, bind CENP-C to promote its correct localization [71]. A more recent study demonstrated that the depletion of endogenous CENP-A could be rescued by a mutant CENP-A in which serine 7 was replaced by a non-phosphorylatable alanine, with no significant appearance of lagging chromosomes, misaligned chromosomes, micronuclei, or altered CENP-C localization. Nonetheless, replacing four serines (including S7, S16, and S18) with non-phosphorylatable alanines or phosphomimetic glutamines in the CENP-A amino-terminal tail only partially rescued the CENP-A depletion in terms of CENP-C recruitment and cell proliferation [69]. The results of these manipulations are similar to the outcome of removing the entire CENP-A N terminus [69,71]. Therefore, CENP-A amino tail phosphorylation seems to be necessary for its centromere incorporation and for proper mitotic completion, but the specific contribution of S7 phosphorylation is still controversial.

Furthermore, the exogenous expression of a mutant CENP-A, in which serines 16 and 18 were replaced by non-phosphorylatable alanines, caused the appearance of lagging and misaligned chromosomes in human dividing cells [74]. Conversely, CENP-A bearing the individual replacement of S16 or S18 for alanine had no significant effects on chromosome segregation [48,74]. Further evidence suggested that S16ph and S18ph promote centromeric chromatin higher-order organization. In another study, S18ph, via the CDK2/cyclin E complex, was shown to inhibit premature CENP-A centromeric deposition. This modification prevents CENP-A’s association with HJURP, restricting CENP-A centromeric incorporation to late mitosis/early G1. Moreover, the S18D phosphomimetic mutation of CENP-A hinders its deposition and promotes chromosome instability and tumorigenesis [48]. Together, these data suggest that a single PTM can have different functions along the CENP-A cycle.

Serine 18 is not the only “stop sign” that prevents premature CENP-A centromeric incorporation. It has been shown that serine 68 is phosphorylated by CDK1 during mitosis [49]. Some studies suggest that this residue is necessary for CENP-A recognition by HJURP [49,75]. According to one model, S68 phosphorylation disrupts this interaction during mitosis, and S68 is then removed by PP1γ at the end of this phase, licensing CENP-A’s association with HJURP and, consequently, promoting its opportune deposition at centromeres [49]. However, conflicting evidence states that S68 phosphorylation is neither sufficient nor necessary for CENP-A loading, centromere function, or cell viability [76]. Interestingly, S68 is evolutionarily conserved in most eukaryotes [49]. Replacing the corresponding amino acid in mice (i.e., S62) for glutamine (which precludes an interaction between CENP-A and a cleft in HJURP) or glutamate (which partially mimics phosphorylated serine 68) has been shown to impair CENP-A deposition and cell viability, and to cause embryonic lethality [75,77].

Moreover, the CENP-A-HJURP interaction is mediated by distinct binding inter-faces in different species. For instance, human HJURP seems to rely heavily on CENP-A’s CENP-A targeting domain (CATD) to recognize this histone while, in chickens, A59 (which corresponds to human S68 but is non-phosphorylatable) is critical for CENP-A-HJURP interaction [78].

Therefore, although it has been established that the essentiality of S68 differs between species, whether its PTMs are required for long-term centromere function is still a matter of debate [49,76,79].

CENP-A is also ubiquitinated on lysine 124 via the CUL4 E3 ubiquitin ligase in complex with COPS8 and RBX1 during mitosis/G1 [80]. This HPTM was shown to be necessary for the correct incorporation of CENP-A, since it promotes its interaction with its chaperone HJURP. Furthermore, substituting lysine 124 by arginine hinders CENP-A centromeric recruitment [80]. As in the case of CENP-A-S68ph, the contribution of K124 ubiquitination to long-term centromere function is controversial [76]. To some extent, this debate has revolved around the methods used by two different research teams [80,81,82]

An alternative hypothesis to the requirement of this HPTM for CENP-A centromeric incorporation states that its recruitment depends exclusively on residues located in the CATD, which comprises amino acids 75–114 [83]. However, only future research will clarify whether CENP-A residues that have recently drawn attention, along with their respective PTMs, play an essential part in the correct CENP-A deposition and centromeric function.

CENP-A methionine 1 is excised by methionine aminopeptidase, similarly to H3 (the amino acid count is displaced, leaving glycine 2 in position 1). This modification leaves glycine 1 exposed, resulting in trimethylation by NMRT [74,84]. Although CENP-A-G1me3 can be detected in the prenucleosomal pool, it is more abundant in nucleosomal CENP-A. Furthermore, this HPTM is required to recruit CENP-I and CENP-T and, hence, correct kinetochore function [84].

On the other hand, Cse4, the CENP-A homolog in S. cerevisiae, has been shown to suffer sumoylation, mediated by the E3 SUMO ligases Siz1 and Siz2 [85,86]. Recent work has demonstrated that the sumoylation of lysines 215 and 216 increases both the centromeric and non-centromeric deposition of Cse4, since they promote its interaction with the histone chaperones CAF-1 and Scm3 (Scm3 is the yeast homolog of human HJURP) [85,87]. Furthermore, replacing Cse4 K215 and 216 for arginines or alanines leads to chromosome mis-segregation [87]. Cse4 is also sumoylated on lysine 65, as a mechanism to prevent its incorrect localization [88]. This modification is recognized by the ubiquitin E3 ligases Slx5 and Psh1, which target mislocalized or overexpressed Cenh4 for proteasome-mediated degradation [85,88]. Interestingly, Slx5 has a mammalian ortholog named RNF4, which is associated with the regulation of chromosome segregation in human cells [88,89]. Therefore, it will be interesting to determine whether mammalian CENP-A function is also regulated by sumoylation systems, similar to yeast Cse4.

Taken together, the experimental data show that CENP-A HPTMs are essential for proper chromosome segregation since they have essential roles in promoting CENP-A loading, restricting its deposition along the cell cycle, and correct kinetochore assembly. Nevertheless, the elucidation of the exact function of some of these marks will require further research. Moreover, the data generated by the ongoing controversies regarding the relevance of CENP-A PTMs outside of the CATD underscore the sensitivity of the CENP-A incorporation control systems to experimental manipulation, as well as the importance of comparative biology (such as in the case of Ser68) in the field. These aspects should be taken into consideration in future experimental settings.

## 4. HPTMs on SAC Activity

We reviewed that HPTMs result in essential modifications in the centromere structure and kinetochore function. Therefore, these modifications are expected to have implications for other downstream mechanisms involved in cell division. One is the spindle assembly checkpoint (SAC). This mechanism prevents chromosome mis-segregation and aneuploidy in mitotic cells by delaying anaphase until all chromosomes are attached to the mitotic spindle and the tension generated by poleward forces in these attachments is correct [90].

One HPTM that has been implicated in the regulation of SAC is the methylation of H3K4. It acts as a negative regulator of SAC through direct binding to the closed conformation of the mitotic arrest deficient protein 2 (C-Mad2), an important player of SAC that by interacting with Cdc20, prevents the activation of APC (anaphase promoter complex). Schibler et al. suggested that the binding to H3K4me could limit C-Mad2 interactions with Cdc20, as part of the normal regulation of SAC. On the contrary, the loss of H3K4me affected Mad2 localization in dividing cells, which in turn conferred resistance to benomyl in Saccharomyces cerevisiae due to a missing functional SAC [91]. Accordingly, Beilharz et al. also found that a loss of H3K4 methylation caused benomyl resistance in several strains of Saccharomyces cerevisiae and that this might be related to an increased expression of alpha tubulin, which in turn could enhance microtubule stability or spindle formation [92]. The authors suggested that since the active benomyl component MBC binds to microtubules, increased tubulin levels may directly contribute to benomyl resistance. Alternatively, another possibility is that abnormally enhanced microtubule stability could affect the tension in the mitotic spindle. The lack of tension generated by poleward forces in the spindle activates the SAC to prevent chromosome mis-segregation in the mitotic cell. In this regard, Luo et al. found that a small region of H3, K37-G44, is necessary to interact with and recruit Shugoshin (SGO1) to the pericentromeric region in *Saccharomyces cerevisiae*. Since SGO1 plays a key role in mitotic tension sensing, this interaction is essential to activate the SAC under a lack of tension in the mitotic spindle. The authors do not rule out the possibility of a post-translational modification of H3 responsible for the interaction with SGO1, but further exploration is needed [93]. What remains to be seen, either in yeast or mammal cells, is whether any specific methylated state of H3K4 could inhibit SAC by interacting with C-Mad2 or promote SAC by recruiting SGo1 to sense the tension between kinetochores and the spindle. Nonetheless, the evidence presented here uncovers an interesting regulating role of an HPTM in the SAC activity and the tension sensing in the mitotic spindle, highlighting the importance of H3K4me in preventing chromosome mis-segregation and possibly chromosome instability.

## 5. Environmental Factors, Histone Post-Translational Modifications, and Genome Maintenance

We reviewed that certain HPTMs play an essential role in proper chromosome condensation and centromere function during mitosis. Therefore, the abnormal alteration of these HPTMs or their regulators brings upon the cell severe consequences that can, in some cases, lead to genomic instability. Moreover, it is known that HPTMs and histone variants are regulated by intra and extracellular factors and by environmental factors. Consequently, the environment is also implicated in chromatin organization, gene expression, chromosome segregation, and a rise in genomic aberrations [94]. Here, we list some environmental factors that can modify histone marks related to chromosome structures important in mitosis.

Arsenic is a pollutant metalloid widely distributed in nature and ubiquitous in soil, water, and air. Humans are chronically exposed to arsenic compounds through contaminated food and drinking water [95,96]. In nature, it can be found in organic or inorganic form, with the latter being the most toxic. The metabolism of inorganic arsenic gives rise to different methylated forms, of which dimethylarsinous acid (DMAIII) and monomethylarsonous acid (MMAIII) are the most toxic in vitro studies [97,98]. Arsenic has multiple cellular effects; it inhibits DNA repair, increases cellular oxidative stress, promotes apoptosis, provokes mitotic alterations, and modifies epigenetic marks [99]. It has been shown that arsenic causes an increase in mitosis length, the formation of chromosomal lags, and the generation of aneuploidies in vitro [95,100,101]. The consequences of cellular exposure to arsenic depend on its chemical species and dose, e.g., sodium arsenite in high doses (greater than 5 micromolar) causes cell death after mitotic arrest instead of only delaying mitosis [95,101].

Although some of the effects of arsenic in mitosis have been associated with its binding to tubulin [102], the formation of the mitotic spindle [103], and the inhibition of proteins such as Plk1 [95], changes in histone marks could alter the mechanisms of condensation and centromere functioning, provoking the mitotic alterations observed in arsenic-treated cells. For example, Suzuki et al. showed that treatment with DMAIII results in the increased phosphorylation of H3S10, a mark associated with chromosome condensation. Furthermore, treatment with DMAIII affected the CPC’s proper location and chromosome segregation [104]. Thus, alteration of this histone mark could inhibit adequate chromosome condensation and segregation. As previously mentioned, the phosphorylation of H3S10 allows the recruitment of Hst2p, which deacetylates the H4K16 residue by promoting chromosome compaction. Arsenic treatment decreases the overall levels of histone acetylation [105,106,107] and H4K16ac by binding to and inhibiting hMOF (the histone acetylase enzyme responsible for the acetylation of H4K16) [106,108]. Because this alteration was evaluated in the interphase, it will be relevant to determine whether arsenic also modifies the acetylation of H4K16 in mitosis and the consequences it may have on chromosome segregation. As already stated, the presence of H3K4me2 and H2Bub1 modifications are significant for CENP-A recruitment and centromere maintenance. In this regard, it has been shown that cellular exposure to arsenic causes an increase in global H3K4me2 and H3K4me3 [109,110,111]. Although the centromeric region has a significant level of H3K4me2, it also has low levels of H3K4me3, so the increase in the latter mark could modify the structure of centromeric chromatin and alter the recruitment of CENP-A.

On the other hand, treatment with arsenic for long periods has the opposite effect, causing a decrease in both H3K4me2 and H3K4me3, the latter being associated with an increase in KDM5 (a histone demethylase) [112]. In C. elegans, it was shown that arsenic treatment promotes a transgenerational reduction in H3K2me2 levels associated with a decrease in spr-5 expression (which codes for a histone demethylase that removes the H3K4me2 mark) [109]. On the other hand, arsenic can bind to the RNF20 protein (the enzyme that generates histone H2B ubiquitination) and inhibit its function, causing a decrease in H2Bub1 levels [113]. As the overall modification of H3K4me2 and H2Bub1 by exposure to arsenic has been evaluated, it will be relevant to define whether these alterations are also present at the centromeric level. It is also essential to determine whether these changes are responsible for specific defects during chromosome segregation.

Nickel (Ni) is a metal found in the environment and originated from the erosion of rocks and volcanic origin. Most of the Ni has an anthropogenic origin due to fossil fuels and industrial processes [114]. Ni is deposited in the soil and is also found in the atmosphere. It can be found as elemental nickel (Ni), nickel oxide (NiO), nickel chloride (NiCl2), and nickel sulfate (NiSO4), among others [115]. Different studies have shown that exposure to Ni causes alterations in chromosome segregation. The treatment of cell cultures with Ni promotes an increase in cells with kinetochore-positive micronuclei [115,116,117]. Moreover, an increase in aneuploid cells and alterations during anaphase have been found after treatment with Ni compounds [116,118].

In various models, it has been shown that Ni can modify histone marks, some of which may be related to mitotic chromosome formation and functioning. In vitro assays have demonstrated that Ni promotes chromatin condensation independently of the cellular machinery. Moreover, Ni treatment is associated with DNA methylation and gene silencing [119,120,121]. As previously mentioned, H3S10ph is an important mark for chromosome compaction. Cells treated with Ni increase the overall levels of H3S10ph [122]. However, the increase in H3S10ph was observed only at the interphase, so it is unclear whether this alteration can affect mitotic chromosome compaction. The role of H3S10ph in the compaction of chromosomes is also associated with the deacetylation of histone H4. Ni diminishes global acetylation of H4 [123,124]. It has been proposed that the inhibition of H4ac is due to the formation of ROS caused by Ni [125]. However, it has also been associated with the direct binding of Ni to histone H4, which prevents its binding to HATs [126]. Moreover, Ni has been shown to decrease local acetylation of H3 and increase H3K9me2 and H3K9me [127,128]. Finally, Ni also increases global levels of H2Aub and H2Bub by inhibiting the deubiquitination of both histones [129]. In most of these studies, changes in histone marks have been studied globally, so it will be relevant to define whether these alterations are present in specific loci and if Ni alters particular marks. It will also be essential to know if modifications in histone marks are associated with defects in segregation caused by Ni and if these marks are specifically modified during mitosis.

In addition to Ni, other metals can modify histone marks and chromosome segregation. Cadmium, for example, causes the formation of aneuploidies in vitro and changes the methylation patterns of DNA and histones [130]. Cadmium also increases H3K4me3 and H3K9Me2 by inhibiting the demethylases that remove these marks [131].

On the other hand, ambient particulate matter (PM) can modify both histone marks and chromosome segregation. Exposure to soil and road dust decreased the expression of Suv39h1 (a histone methyltransferase responsible for H3K9 methylation) with a concomitant increase in centromeric satellite DNA expression. As previously mentioned, the expression of these sequences is tightly associated with CENP-A incorporation. Accordingly, the authors of this work observed chromosome aberrations under soil dust exposure in vitro [132]. Airborne particulate matter also modifies histone mark patterns. PM10 (particles with an aerodynamic diameter smaller than 10 μm) exposure promotes acetylation of histone H4 by increasing HAT activity [133]. Moreover, PM10-exposed human cells showed an increase in micronucleated and trinucleated cells [134]. However, particles obtained from different regions or different times of the year may have a different composition. The same PM10 sample must be used for experiments to determine whether PM10 exposure can alter histone marks and chromosome segregation.

## 6. Conclusions

HPTMs and the deposition of histone variants play a crucial role in distinct stages of mitosis, starting from chromatin condensation, centromere formation, kinetochore assembly, and dynamics of the mitotic spindle during cell division. Therefore, the deregulation of HPTMs can lead to chromosome mis-segregation and errors in cell division. It has been suggested that the cell responds to environmental stimuli and challenges through HPTMs and the deposition of histone variants [135]. Therefore, it is expected that chromatin condensation and mitosis regulation might be disturbed by environmental insults. Exposure to genotoxic and radiation agents is related to changes in certain HPTMs and the deposition of histone variants and is associated with deleterious effects such as DNA damage, aneuploid-associated syndromes, and chromosome instability, a well-known hallmark of cancer. It has been suggested that certain environmental factors are associated with cancer, but only a few examples are well explained, and their carcinogenic mechanisms are known in detail [135,136]. Plenty of pollutant agents are widely spread in our environment due to the current human lifestyle and industrial and transport activities. Their impact on distinct cell functions and pathways needs to be addressed. The deregulation of mitotic HPTMs will certainly uncover the carcinogenic potential of many of those pollutant agents and help societies and decision-makers to search for healthier and safer alternatives.

## Data Availability

Not applicable.
